# Single-cell transcriptomics reveals multiple neuronal cell types in human midbrain-specific organoids

**DOI:** 10.1007/s00441-020-03249-y

**Published:** 2020-07-31

**Authors:** Lisa M. Smits, Stefano Magni, Kaoru Kinugawa, Kamil Grzyb, Joachim Luginbühl, Sonia Sabate-Soler, Silvia Bolognin, Jay W. Shin, Eiichiro Mori, Alexander Skupin, Jens C. Schwamborn

**Affiliations:** 1 Luxembourg Centre for Systems Biomedicine (LCSB), Developmental and Cellular Biology, University of Luxembourg, Belvaux, Luxembourg; 2 Department of Future Basic Medicine, Nara Medical University, Kashihara, Nara, Japan; 3 Division of Genomic Technologies, RIKEN Center for Life Science Technologies, Yokohama, Kanagawa Japan; 4 University California San Diego, La Jolla, CA USA

**Keywords:** Neural stem cells, Midbrain organoids, Neuronal subtypes, Single-cell RNA sequencing, Electrophysiological activity

## Abstract

Human stem cell-derived organoids have great potential for modelling physiological and pathological processes. They recapitulate in vitro the organization and function of a respective organ or part of an organ. Human midbrain organoids (hMOs) have been described to contain midbrain-specific dopaminergic neurons that release the neurotransmitter dopamine. However, the human midbrain contains also additional neuronal cell types, which are functionally interacting with each other. Here, we analysed hMOs at high-resolution by means of single-cell RNA sequencing (scRNA-seq), imaging and electrophysiology to unravel cell heterogeneity. Our findings demonstrate that hMOs show essential neuronal functional properties as spontaneous electrophysiological activity of different neuronal subtypes, including dopaminergic, GABAergic, glutamatergic and serotonergic neurons. Recapitulating these in vivo features makes hMOs an excellent tool for in vitro disease phenotyping and drug discovery.

**Electronic supplementary material:**

The online version of this article (10.1007/s00441-020-03249-y) contains supplementary material, which is available to authorized users.

## Introduction

Current in vitro approaches to model physiology and pathology of human neurons are mainly based on pure cultures of neurons grown under 2D conditions. It has been shown that the differentiation potential of human induced pluripotent stem cells (iPSCs) provides a unique source of different neural cell types (Takahashi and Yamanaka 2006). Until now, many protocols for generating iPSC-derived neural cultures have been described. The resulting cell culture monolayers have been proven as useful tools to study disease mechanisms and to identify potential neuroprotective compounds (Nguyen et al. 2011; Cooper et al. 2012; Sánchez-Danés et al. 2012; Reinhardt et al. 2013b; Ryan et al. 2013). However, these culture conditions do not recapitulate several characteristics, which are relevant to the human brain, like cyto-architecture or complex cell-cell interactions. This may result in inaccurate modelling of the human brain (patho-)physiology with the consequence that candidate compounds might prove efficacy in 2D in vitro studies but are ineffective in clinical trials or vice versa (Abe-Fukasawa et al. 2018). The recent establishment of new 3D neuronal cell culture models has contributed to mimic key aspects of human brain development (Lancaster et al. 2013; Tieng et al. 2014; Muguruma et al. 2015; Jo et al. 2016; Qian et al. 2016; Monzel et al. 2017). Studies using human cerebral brain organoids have shown the acquisition of neuronal maturity and network activity (Quadrato et al. 2017; Matsui et al. 2018). Their complex, multicellular architecture enables the study of neuronal diseases and has already led to novel insights on, e.g. Zika virus–induced microcephaly (Ming et al. 2016; Qian et al. 2017). Besides this unique in vitro disease modelling potential, human brain organoids provide a platform for advanced drug screening (Kelava and Lancaster 2016; Di Lullo and Kriegstein 2017). In this study, we focused on a detailed characterization of the different neuronal subtypes in human midbrain-specific organoids (hMOs). With single-cell transcriptome analysis, we examined the presence of different neuronal subtypes, and subsequently studied the effect of chemical compounds on the electrophysiological activity of the neuronal network. Our findings demonstrate that hMOs contain, beside dopaminergic neurons, other neuronal subtypes including GABAergic, glutamatergic and serotonergic neurons. hMOs showed essential neuronal functional properties during the course of differentiation, like synapse formation and spontaneous electrophysiological activity. These features indicate that hMOs recapitulate specific characteristics of functional human midbrain tissue, thus making them a valuable resource for in vitro disease modelling and drug discovery.

## Material and methods

### Data availability

The data that support the findings of this study, including the original single-cell RNA Sequencing data, are publicly available at this doi: www.doi.org/10.17881/lcsb.20190326.01.

Furthermore, a previous version of this manuscript is available as pre-print under: 10.1101/589598.

### Pluripotent stem cell culture

hiPSC lines were provided by Bill Skarnes, Wellcome Trust Sanger Institute (iPSC Bill), Alstem (iPS15, derived from human peripheral blood mononuclear cells, episomal reprogrammed) or previously described in Reinhard et alia (Reinhardt et al. 2013b). The cells were cultured on Matrigel-coated (Corning, hESC-qualified matrix) plates, maintained in Essential 8 medium (Thermo Fisher Scientific) and cultured with and split 1:6 to 1:8 every 4 to 5 days using Accutase (Sigma). Ten μM ROCK inhibitor (Y-27632, Abcam) was added to the media for 24 h following splitting.

### Derivation of midbrain floorplate neural progenitor cells

The derivation and maintenance of midbrain floorplate neural progenitor cells (mfNPCs) has been described previously (Smits et al. 2019).

In brief, embryoid bodies (EBs) were formed with 2000 iPSCs each, using AggreWell 400 (Stemcell Technologies). The cells were cultured in Knockout DMEM (Invitrogen) with 20% Knockout Serum Replacement (Invitrogen), 100-μM beta-mercaptoethanol (Gibco), 1% nonessential amino acids (NEAA, Invitrogen), 1% penicillin/streptomycin/glutamine (Invitrogen), freshly supplemented with 10-μM SB-431542 (SB, Ascent Scientific), 250-nM LDN-193189 (LDN, Sigma), 3-μM CHIR99021 (CHIR, Axon Medchem), 0.5-μM SAG (Merck) and 5-μM ROCK inhibitor (Sigma). After 24 h, EBs were transferred to a non-treated tissue culture plate (Corning). On day two, medium was replaced with N2B27 medium consists of DMEM-F12 (Invitrogen)/Neurobasal (Invitrogen) 50:50 with 1:200 N2 supplement (Invitrogen), 1:100 B27 supplement lacking vitamin A (Invitrogen) with 1% penicillin/streptomycin/glutamine, supplemented with 10-μM SB, 250-nM LDN, 3-μM CHIR and 0.5-μM SAG. On day four and six, medium was exchanged with the same but including 200-μM ascorbic acid (AA, Sigma). On day eight, EBs with neuroepithelial outgrowth were triturated into smaller pieces and diluted in a 1:10 ratio. For following passages, 1× TrypLE Select Enzyme (Gibco)/0.5-mM EDTA (Invitrogen) in 1× PBS was used and 10,000 to 20,000 cells per 96-well ultra-low attachment plate (round bottom, Corning) were seeded. The cells were always kept under 3D culture conditions and from passage 1 on cultured in N2B27 medium freshly supplemented with 2.5-μM SB, 100-nM LDN, 3-μM CHIR, 200-μM AA and 0.5-μM SAG. After every cell split, the ultra-low attachment plate was centrifuged for 3 min at 200×*g* to assure the aggregation of single cells at the bottom of the well. Additionally, a 5-μM ROCK inhibitor was added. The cells were split every 7 to 14 days and the medium was changed every third day. After four to five passages, mfNPCs were used as a starting population for hMOs.

### Generation of midbrain-specific organoids

To start the generation of hMOs, 3000 cells per well were seeded to an ultra-low attachment 96-well round bottom plate, centrifuged for 3 min at 200×*g* and kept under maintenance conditions for 7 days. LDN and SB were withdrawn of mfNPC expansion medium and after three additional days, the concentration of CHIR was reduced to 0.7 μM. On day nine of differentiation, medium was changed to neuronal maturation N2B27 medium including 10-ng/ml BDNF (Peprotech), 10-ng/ml GDNF (Peprotech), 200-μM AA (Sigma), 500-μM dbcAMP (Sigma), 1-ng/ml TGF-β3 (Peprotech), 2.5-ng/ml ActivinA (Life Technologies) and 10-μM DAPT (Cayman). The organoids were kept under static culture conditions with media changes every third day for 35 or 70 days. Detailed information about the generation of hMOs has been published recently (Smits et al. 2019).

### Immunofluorescence

hMOs were fixed with 4% PFA overnight at 4 °C and washed 3× with PBS for 15 min. After treatment, they were embedded in 3–4% low melting point agarose in PBS. The solid agarose block was sectioned with a vibratome (Leica VT1000s) into 50 or 70-μm sections. The sections were blocked on a shaker with 0.5% Triton X-100, 0.1% sodium azide, 0.1% sodium citrate, 2% BSA and 5% normal goat or donkey serum in PBS for 90 min at RT. Primary antibodies were diluted in the same solution but with only 0.1% Triton X-100 and were applied for 48 h at 4 °C.

After incubation with the primary antibodies (Supplementary Table 2), sections were washed 3× with PBS and subsequently blocked for 30 min at RT on a shaker. Then sections were incubated with the secondary antibodies in 0.05% Tween-20 in PBS for 2 h at RT and washed with 0.05% Tween-20 in PBS and Milli-Q water before they were mounted in Fluoromount-G mounting medium (Southern Biotech).

STAINperfect Immunostaining Kit (ImmuSmol) was used according to manufacturer’s protocol to detect dopamine, serotonin, GABA and L-glutamine. Nuclei were counterstained with Hoechst 33342 (Invitrogen).

For qualitative analysis, three randomly selected fields per organoid section were acquired with a confocal laser scanning microscope (Zeiss LSM 710) and images were further processed with OMERO Software. Three-dimensional surface reconstructions of confocal z-stacks were created using Imaris software (Bitplane).

### Quantitative image analysis

Immunofluorescence 3D images of hMOs were analysed in Matlab (Version 2017b, Mathworks). The in-house developed image analysis algorithms automate the segmentation of nuclei, astrocytes and neurons with structure-specific feature extraction. The image preprocessing for the segmentation of nuclei was computed by convolving the raw Hoechst channel with a Gaussian filter. By selecting a pixel threshold to identify apoptotic cells, a pyknotic nuclei mask was identified and subtracted from the nuclei mask.

For the segmentation of neurons, a median filter was applied to the raw TUJ1 channels. The expression levels were expressed in two ways as follows: (i) positive pixel of the marker, normalized by the pixel count of Hoechst; (ii) cells positive for a marker expressed as a percentage of the total number of cells. In this latter case, the nuclei were segmented and a watershed function was applied. Considering the high cell density of the specimens, steps to ensure high quality in the segmentation process were implemented and structures with a size higher than 10,000 pixels were removed (this indicated incorrected segmentation, e.g. clumps). In the nuclei successfully segmented as a single element, a perinuclear zone was identified. In case the marker of interest was positive in at least 1% of the perinuclear area, the corresponding cell was considered as positive.

### Single-cell RNA sequencing using droplet-sequencing (Drop-Seq)

Single-cell RNA sequencing (scRNA-seq) data were generated using the Droplet-Sequencing (Drop-Seq) technique (Macosko et al. 2015) as described previously (Walter 2019). In this work, we performed scRNA-seq of hMOs derived from hiPSC line H4 (see Supplementary Table 1). For each time point, 35 days and 70 days after dopaminergic differentiation, we pooled and analysed 30 hMOs each.

### Pre-processing of the digital expression matrices from scRNA-seq

The result of the Drop-Seq scRNA-seq pipeline and subsequent bioinformatics processing is a digital expression matrix (DEM) representing the number of mRNA molecules captured per gene per droplet. Here, we obtained two DEMs, one corresponding to 35-day hMOs and the other to 70-day hMOs. After quality cut based on knee plots, we retained for each sample 500 cells with the highest number of total transcripts measured and performed normalization of the DEM separately. Finally, the two DEMs were merged for the comparison analysis of the two time points based on 24,976 expressed genes in 1000 cells. The data was analysed by our customized Python analysis pipeline (Python version 3.6.0, with anaconda version 4.3.1) including dimensionality reduction by t-distributed stochastic neighbourhood embedding (t-SNE) (van der Maarten and Hinton 2008) and differential gene expression analysis.

### Analysis of differentially expressed genes from scRNA-seq data

To determine which and how many genes were differentially expressed between 35-day and 70-day hMOs, we applied a one-way ANOVA test, a one-way ANOVA test on ranks (Kruskal-Wallis test), and a Mutual Information based test. The minimum *p* value obtained for each gene across these three tests was retained and statistical significance was set to *p* < 0.01 after Bonferroni correction for multiple hypothesis testing of differentially expressed genes (DEGs).

### Cumulative gene expressions from scRNA-seq data

From literature, we extracted cell type–specific gene lists (Supplementary Table 3) for stem cells, neurons and neuronal subtypes (dopaminergic, glutamatergic, GABAergic and serotonergic neurons) (Reinhardt et al. 2013a; La Manno et al. 2016; Cho et al. 2017). Note that not all genes listed therein have been measured in our dataset; these were highlighted in Supplementary Table 3.

For each list, we defined a score, which we refer to as cumulative gene expression, computed as the sum of the expression of the corresponding genes from normalized DEM for each cell. Since the expression levels were measured at single cell level, we can consider the cells’ distributions across the cumulative genes expression scores (Fig. 2a). These histograms exhibit the cumulative gene expression scores normalized to their maxima on the horizontal axis. Thus, on the horizontal axis, a value of 1 corresponds to the maximal cumulative gene expression for one list of genes, while 0 corresponds to no expression of any genes from that list. The vertical axis exhibits the number of cells falling into the corresponding bin of the histogram. In each subpanel, the distributions for day 35 and for day 70 are shown. Population differences were assessed by Z-test of the means with Bonferroni correction.

### Gene-gene correlations from scRNA-seq data

**Fig. 1:**
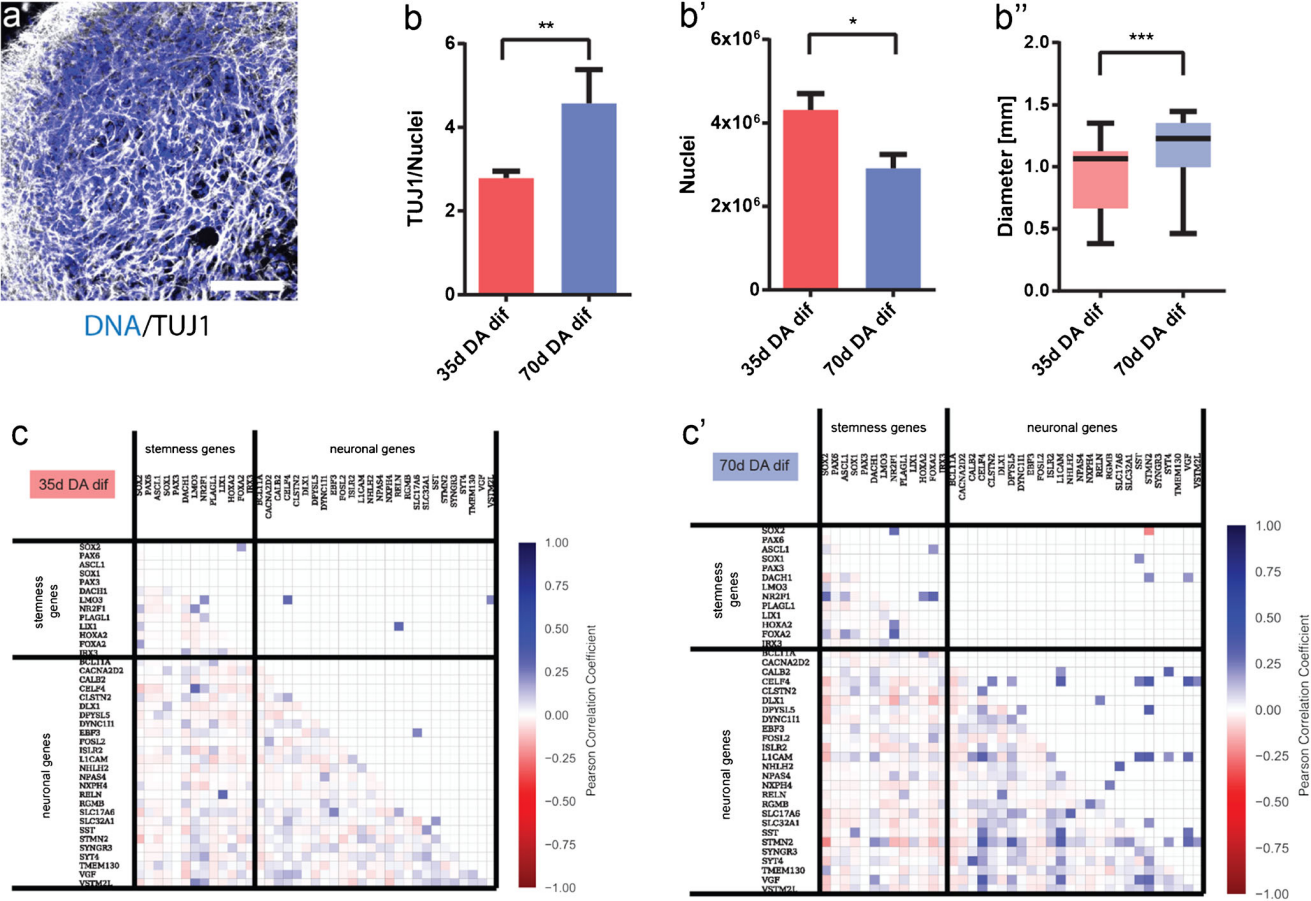
Identification of neuronal population in midbrain-specific organoids. (**a**) Immunohistological staining of TUJ1 expressing neurons in 35-day organoid sections (50-μm thickness, scale bar 100 μm). (**b**) The ratio of TUJ1 positive pixels normalized against Hoechst (35 days *n* = 59, 70 days *n* = 48). (**b’**) Quantification of Hoechst positive pixel (35 days *n* = 22, 70 days *n* = 29). (**b”**) Average size of four different organoid lines. Whiskers present minimum and maximum (35 days *n* = 21, 70 days *n* = 44). Data presented as mean ± SEM. (**c**) Gene-gene correlation matrices, for genes at day 35 (**c**), and day 70 (**c’**)

From the scRNA-seq data, we also computed gene-gene Pearson correlation coefficients for stemness-specific and neuron-specific genes. Analysis was performed independently for the two samples (35-day DA dif and 70-day DA dif) resulting in two correlation matrices (Fig. 1c).

In the lower triangular matrix, all correlation values are shown, whereas the upper triangular matrix contains only statistically significant correlations (*p* value < 0.05 after Bonferroni correction). For visual clarity, diagonal elements and undetected genes were excluded.

### Fold changes of gene expression from scRNA-seq data

**Fig. 2:**
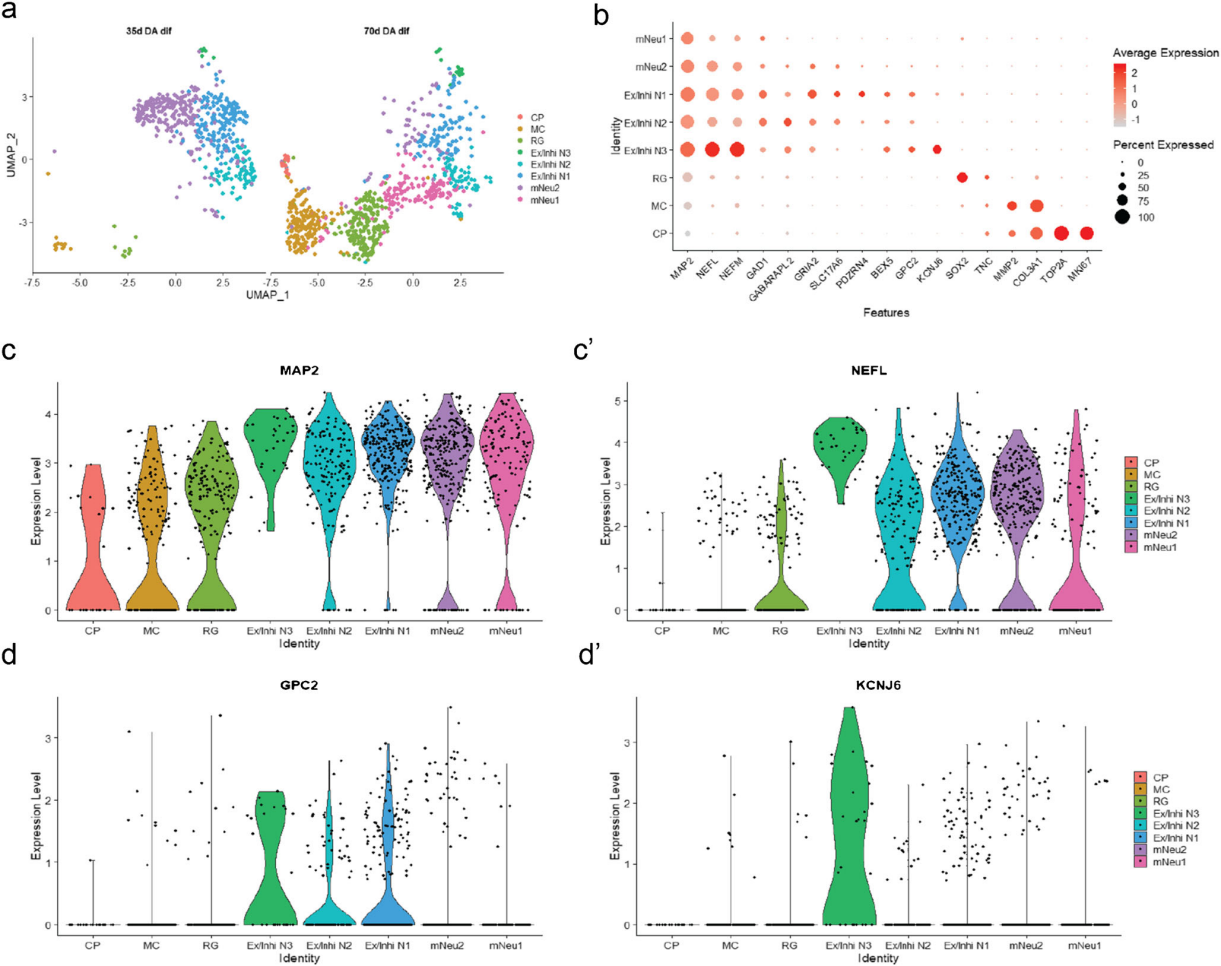
Single-cell RNA sequencing analysis of midbrain-specific organoids. (**a**) Uniform manifold approximation and projection (UMAP) plot shows that the total 1295 cells identified 8 cell populations. Each dot corresponds to a single cell. Cell populations are coloured and annotated based on their expressing genes. CP, cycling progenitors; MC, mesenchymal cells; RG, radial glia cells; Ex/Inhi N, excitatory/inhibitory neurons; mNeu, mature neurons. UMAP plots shows difference in gene expressions between day 35 and day 70. (**b**) Dot plot shows the expression of each cell type–specific genes. (**c**–**d**) Violin plots show the distribution of expression of each marker gene. The mNeu clusters expressed MAP2 (**c**) and NEFL (**c’**), the Ex/Inhi N clusters expressed GPC2 (**d**) and KCNJ6 (**d’**)

For individual genes, we considered the normalized gene expression across cell populations. For each selected gene, we compared its expression within the 35-day cells with the one within the 70-day cells by computing the logarithmic fold change (log2FC). We performed this analysis for the genes specific of neuronal subtypes including glutamatergic neurons, GABAergic neurons and dopaminergic neurons (Fig. 2c–d), where negative values indicate that a gene is less expressed at day 35 than at day 70 and positive numbers the opposite. *p* values are based on Z-test with Bonferroni correction and significance levels correspond to * = *p* value <0.05, ** = *p* value <0.01, *** = *p* value < 0.001, and **** = *p* value < 0.0001. Error bars represent SEM based on the individual sample average and error propagation.

### scRNA-seq data analysis for UMAP plot, dot plot and violin plot

ScRNA-seq data were generated using the Droplet-Sequencing (Drop-Seq) technique (1). After bioinformatics processing, we obtained two digital expression matrices (DEM), corresponding to day 35 and day 70 after differentiation into human midbrain organoids (hMOs).

In an alternative analysis approach, which is independent and complementary to the scRNA-Seq analysis described above, further data processing was performed using the Seurat v.3.0.0 R package (Satija et al. 2015). Cells with more than 4000 or less than 500 detected genes, as well as those with mitochondrial transcripts proportion higher than 7.5% were excluded. We collected a total of 1295 cells (505 cells at day 35 and 790 cells at day 70). The datasets were log normalized and scaled to 10,000 transcripts per cells. The top 2000 highly variable genes for day 35 and day 70 were determined using the variance-stabilizing transformation method. The datasets from day 35 and day 70 were integrated using canonical correlation analysis (CCA) in the Seurat package (Stuart et al. 2019). The datasets were integrated based on the top 30 dimensions from CCA using the Seurat method by identifying anchors and integrating the datasets. The resulting integrated data were scaled and principal component analysis (PCA) was performed. Clustering was performed based on the top 30 principal components (PCs), using the shared nearest neighbour (SNN) modularity optimization with a resolution of 0.8. Cluster identities were assigned based on cluster gene markers as determined by the “FindAllMarkers” function in Seurat and gene expression of known marker genes.

### TEM morphology

Sixty-three-day-old hMO specimens were immersion-fixed in a solution of 2% PFA and 2.5% glutaraldehyde in 0.1-M sodium cacodylate buffer (pH 7.4, Electron Microscopy Sciences, Hatfield, PA) for 3 h, rinsed several times in cacodylate buffer and further post-fixed in 2% glutaraldehyde in 0.1-M sodium cacodylate buffer for 2 h at room temperature on a gentle rotator; fixative was allowed to infiltrate an additional 48 h at 4 °C. Specimens were rinsed several times in cacodylate buffer, post-fixed in 1.0% osmium tetroxide for 1 h at room temperature and rinsed several times in cacodylate buffer. Samples were then dehydrated through a graded series of ethanols to 100% and dehydrated briefly in 100% propylene oxide. Tissue was then allowed to pre-infiltrate 2 h in a 2:1 mix of propylene oxide and Eponate resin (Ted Pella, Redding, CA), then transferred into a 1:1 mix of propylene oxide and Eponate resin and allowed to infiltrate overnight on a gentle rotator. The following day, specimens were transferred into a 2:1 mix of Eponate resin and propylene oxide for a minimum of 2 h, allowed to infiltrate in fresh 100% Eponate resin for several hours, and embedded in fresh 100% Eponate in flat moulds; polymerization occurred within 24–48 h at 60 °C. Thin (70 nm) sections were cut using a Leica EM UC7 ultramicrotome, collected onto formvar-coated grids, stained with uranyl acetate and Reynold’s lead citrate and examined in a JEOL JEM 1011 transmission electron microscope at 80 kV. Images were collected using an AMT digital imaging system with proprietary image capture software (Advanced Microscopy Techniques, Danvers, MA).

### Microelectrode array

The Maestro microelectrode array (MEA, Axion BioSystems) platform was used to record spontaneous activity of the hMOs. A 48-well MEA plate containing a 16-electrode array per well was precoated with 0.1-mg/ml poly-D-lysine hydrobromide (Sigma-Aldrich). Sixty to seventy days old organoids of two different passages were briefly treated for 5 min with 1× TrypLE Select Enzyme, resuspend in 10 μg/ml laminin (Sigma-Aldrich) and placed as a droplet onto the array. After 1 h incubation, neuronal maturation media was added and cells were cultured for 1–2 weeks. Spontaneous activity was recorded at a sampling rate of 12.5 kHz for 5 min at 37 °C over several days. Axion Integrated Studio (AxIS 2.1) was used to assay creation and analysis. A Butterworth band pass filter with 200–3000 Hz cutoff frequency and a threshold of 6× SD were set to minimize both false positives and missed detections. The spike raster plots were analysed using the Neural Metric Tool (Axion BioSystems). Electrodes with an average of ≥ 5 spikes/min were defined as active, for the pharmacological treatment 24 electrodes were analysed. The organoids were consecutively treated with Gabazine, D-AP-5, NBQX (Cayman Chemical, end concentration: 50 mM each), and Quinpirole (Sigma Aldrich, end concentration: 5 μM). To block all neuronal activity and thus verify spontaneous spiking activity of the cells, tetrodotoxin (TTX, Cayman Chemical, 1 μM) was applied at the end. The spike count files generated from the recordings were used to calculate the number of spikes/active electrode/min. Further details regarding the MEA system were previously described (Bardy et al. 2015).

### Statistical analyses

If not stated otherwise, experiments were performed with three independently generated organoid cultures from three different cell lines (*n* = 9). Gaussian distribution was evaluated by performing D’Agostino and Pearson omnibus normality test. In case the data were normally distributed, Grubbs’ test was performed to detect significant outliers. Unpaired *t* test with Welch’s correction or nonparametric Kolmogorov-Smirnov test was performed to evaluate statistical significance. Data are presented as mean ± SEM. The statistical analyses of scRNA-seq data are described in the corresponding sections.

## Results

### Characterization of the neuronal differentiation dynamics in midbrain-specific organoids

Previously, we demonstrated that human iPSC-derived midbrain floor plate neural progenitor cells (mfNPCs) can give rise to 3D human organoids that contain high amounts of dopaminergic neurons (Smits et al. 2019). To have a better insight into the dynamics of the neuronal differentiation, we evaluated TUJ1 staining, as a marker for neuronal differentiation, at two time points during the differentiation of hMOs (Fig. 1 a and b). An in-house developed image analysis algorithm was used to segment Hoechst-positive nuclei and TUJ1-positive neurons to create specific nuclear and neuronal masks. These masks contain all positive pixel counts for Hoechst and TUJ1, respectively.

The TUJ1 signal normalized to the Hoechst signal significantly increased after 70 days compared with 35 days, demonstrating a progressive differentiation into post-mitotic neurons. Whereas, the nuclear marker signal was significantly decreased at 70 days compared with 35 days, which might indicate selection in the cell population, as reported by Suzanne and Steller (2013) (Fig. 1 b and b’). Along with these findings, we observed that the size of the organoids significantly increased during the course of the differentiation. This suggests that the increased TUJ1 volume and organoid size are due to the increased tissue complexity (e.g. neuronal arborisation) within the hMO (Fig. 1b”).

To further characterize the neuronal differentiation dynamics at the gene expression level, we performed scRNA-seq on samples from the two time points mentioned above. The experiments were conducted using the Drop-Seq technique (Macosko et al. 2015), and the standard bioinformatics processing of the data resulted in two sample-specific digital expression matrices (DEM), which were further normalized and merged (see Methods section).

To investigate how the differentiation of precursor cells into neurons evolves over time, we computed the gene-gene correlation for the genes of the neuron-specific list and of the stemness-specific list, altogether. Comparing these two lists, we found that at 35 days there are low values of correlation between genes exclusively specific for neurons or stem cells and also between neuron- and stemness-specific genes (Fig. 1c). Very few of the correlation values are significantly different from zero and were substituted by zeros in the upper triangular matrix (Fig. 1c). While correlations between stemness genes and neuron-stemness correlations at day 70 remain similar to day 35, correlations between neuron-specific genes increased considerably at day 70. This significant increase of neuron-specific gene correlations indicates a higher commitment of the cells towards the neuronal fate at day 70 compared with day 35 and supports the finding of a progressive maturation of post-mitotic neurons (Fig. 1 c and c’).

To visualize the so-obtained high-dimensional single-cell data, we performed dimensionality reduction of the DEM by uniform manifold approximation and projection (UMAP) (van der Maarten and Hinton 2008), where each dot corresponds to a cell (Fig. 2a). After processing, quality control and filtering, we analysed a total of 1295 cells, 505 cells at day 35 and 790 cells at day 70 (Fig. S1 a and b). To identify distinct cell populations based on shared and unique patterns of gene expression, we performed dimensionality reduction and unsupervised cell clustering. All 1295 cells from the two time points were analysed together and plotted onto two uniform manifold approximation and projection (UMAP) plots (Fig. 2a). We identified eight distinct cell populations expressing known markers of major cell types (Fig. 2b). The cell populations comprised five neuronal clusters and three non-neuronal clusters. The five neuron clusters were divided into two mature neuron cluster (mNeu1 and mNeu2) and three excitatory/inhibitory neuron clusters (Ex/Inhi N1, Ex/Inhi N2, Ex/Inhi N3). The mNeu clusters expressed MAP2, NEFL and NEFM (Fig. 2 b, c and c’). The Ex/Inhi N clusters expressed BEX5, GPC2, KCNJ6, PDZRN4, GRIA2, SLC17A6, GAD1 and GABARAPL2 (Fig. 2 b, d and d’). Non-neuronal clusters were divided into radial glia cells (RG), mesenchymal cells (MC) and cycling progenitors (CP) subtypes (Fig. 2 b, S2 a and b). Cell populations distinctly changed between day 35 and day 70 (Fig. 2a). Interestingly the day 70 cell populations showed more non-neuronal clusters.

### Midbrain-specific organoids consist of different neuronal subtypes

From previous studies, we know that hMOs are rich in dopaminergic neurons (Jo et al. 2016; Qian et al. 2016; Monzel et al. 2017; Smits et al. 2019; Kim et al. 2019). We wanted to further explore which other neuronal subtypes develop besides midbrain dopaminergic neurons within the hMOs.

**Fig. 3:**
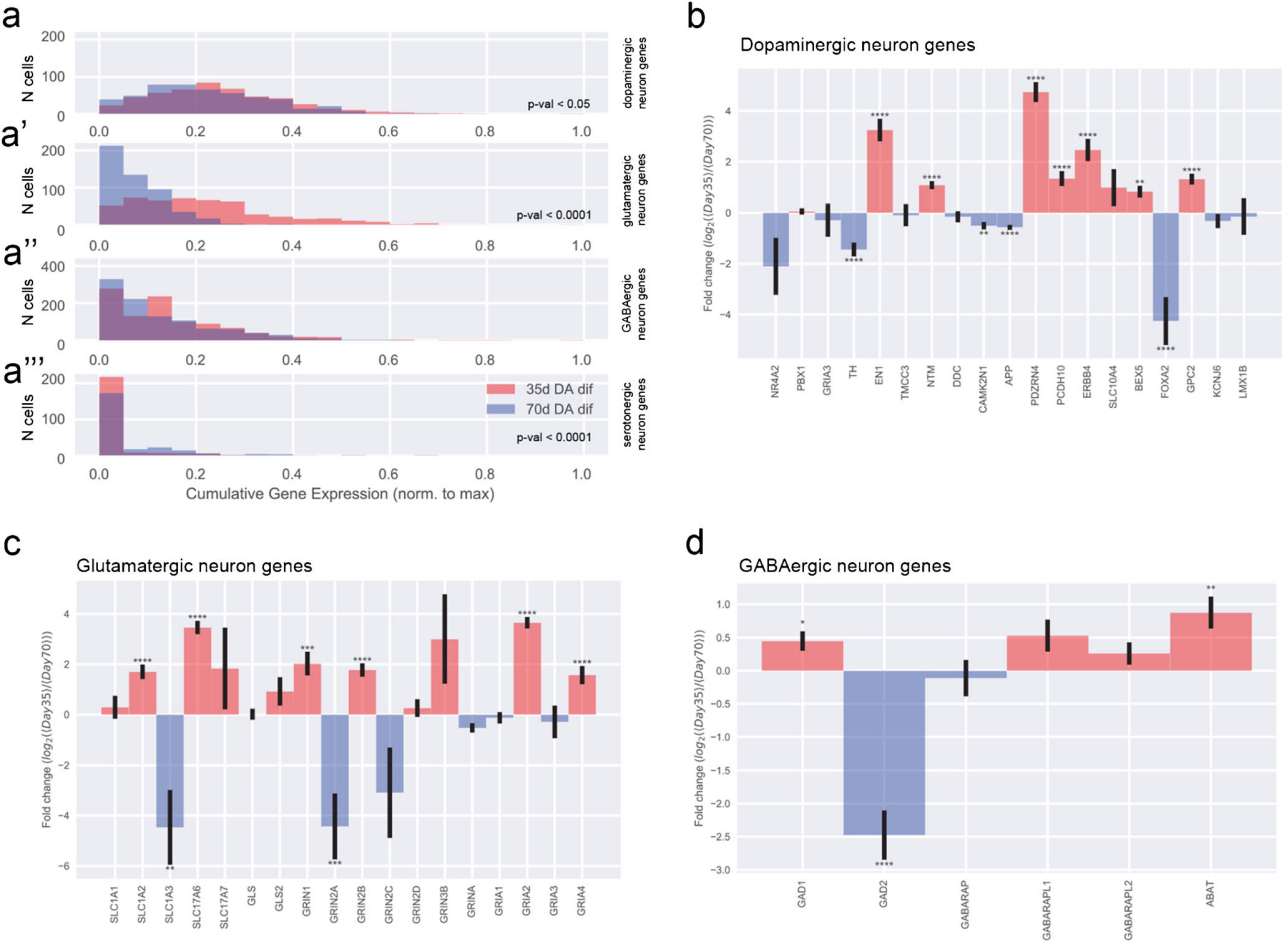
Neuronal subtypes in midbrain-specific organoids. (**a**) Distributions (histograms) of cells across the cumulative gene expression scores, obtained from the lists of genes specific for the main neuronal subtypes present in the organoids, namely, dopaminergic (**a**), glutamatergic (**a’**), GABAergic (**a”**), and serotonergic neurons (**a”’**). (**b**–**d**) Log2 fold-changes between day 35 and day 70 in gene expression for individual genes corresponding to the lists of genes typical of the neuronal subtypes: (**b**) dopaminergic neurons, (**c**) glutamatergic neurons, and (**d**) GABAergic neurons

Therefore, we investigated the expression of genes typical for dopaminergic, glutamatergic, GABAergic and serotonergic neurons by analysing the scRNA-seq data. We plotted the distributions of cells across the cumulative gene expression scores, which were obtained from the lists of genes specific of a neuronal subtype (Fig. 3a–a”’). While the cell distribution over cumulative expression score for GABAergic neurons was very similar between the samples at 35 days and 70 days (Fig. 3a”), we detected statistically significant differences between the distributions of cells over scores for the other three types of neurons. The expression of the selected genes for the glutamatergic and dopaminergic neurons was increased at day 35 compared with day 70, which is consistent with the observations for the neuron specific score (Fig. 2a).

**Fig. 4:**
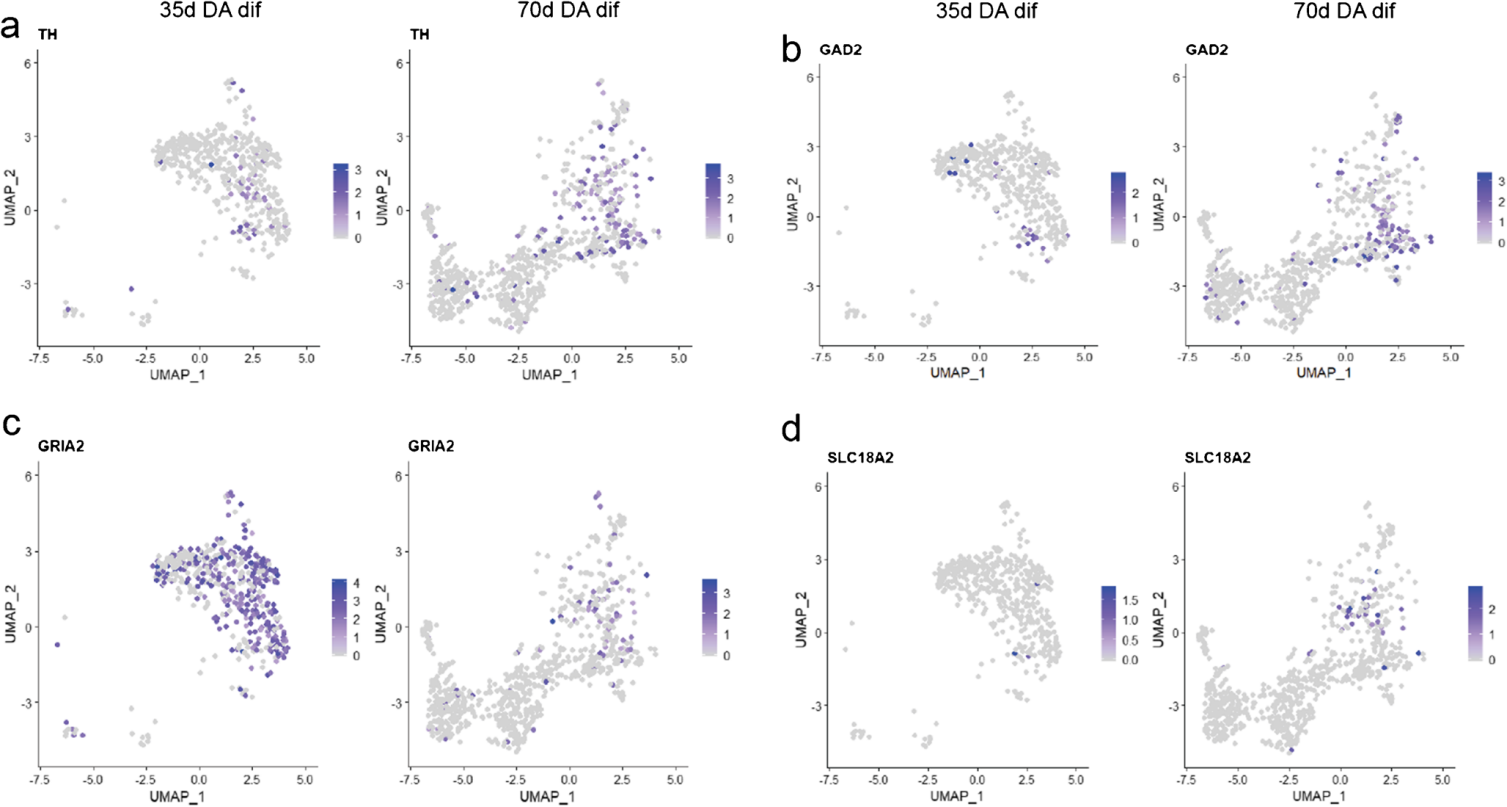
UMAP plot analysis of neuronal subtypes. UMAP plots show the gene expressions at day 35 at day 70 for markers of dopaminergic (**a**), GABAergic (**b**), glutamatergic (**c**), and serotonergic (**d**) neurons. Each dot is coloured according to the expression level

In order to additionally highlight the presence of dopaminergic, glutamatergic, GABAergic and serotonergic neurons, we made use of the UMAP plots and highlighted the expression of marker genes for dopaminergic, glutamatergic, GABAergic and serotonergic neurons (Fig. 4 and S3). In agreement with the fact that the fraction of non-neuronal cells is higher in the day 70 organoids (Fig. 1), the counts for the various neuronal subtypes tend to be higher at day 35.

**Fig. 5:**
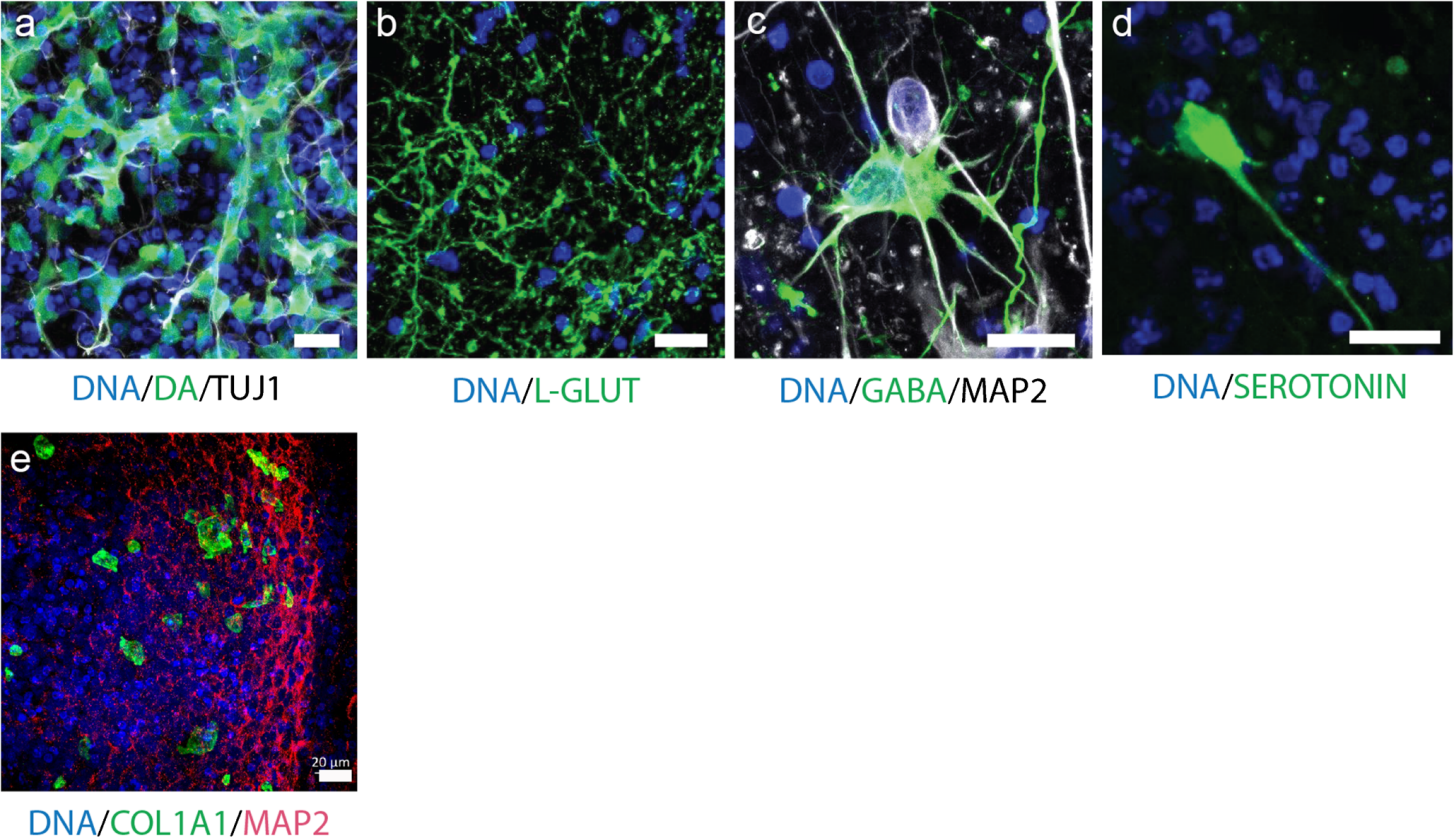
Immunofluorescence staining analysis of neuronal subtypes. (**a**–**d**) Immunohistological staining of organoid sections (50-μm thickness). Detection of the neurotransmitters dopamine (**a**), L-glutamine (**b**), GABA (**c**), and serotonin (**d**). Scale bar is 20 μm. (**e**) Immunohistological staining of organoid sections (70-μm thickness) for the detection of the fibroblast marker COL1A1. Scale bar is 20 μm

To further verify the presence of the addressed neuronal subtypes, we conducted an immunohistochemistry staining for the respective neurotransmitters. This allowed us to robustly detect dopaminergic, glutamatergic and GABAergic neurons as well as even a few serotonergic neurons within hMOs (Fig. 5a–d).

Finally, among the non-neuronal cells, we surprisingly found a cluster of mesenchymal cells (MC in Fig. 1d). A similarly surprising finding has been reported recently for a single-cell RNA sequencing analysis of dopaminergic neurons transplanted in the rodent brain, where fibroblast-like cells were detected (Tiklova et al. 2019). These cells are reported to be positive for the marker COL1A1. Hence, we also stained hMOs with an anti-COL1A1 antibody and indeed we were able to confirm the presence of this cell type (Fig. 5e).

### Midbrain-specific organoids express synaptic proteins

**Fig. 6:**
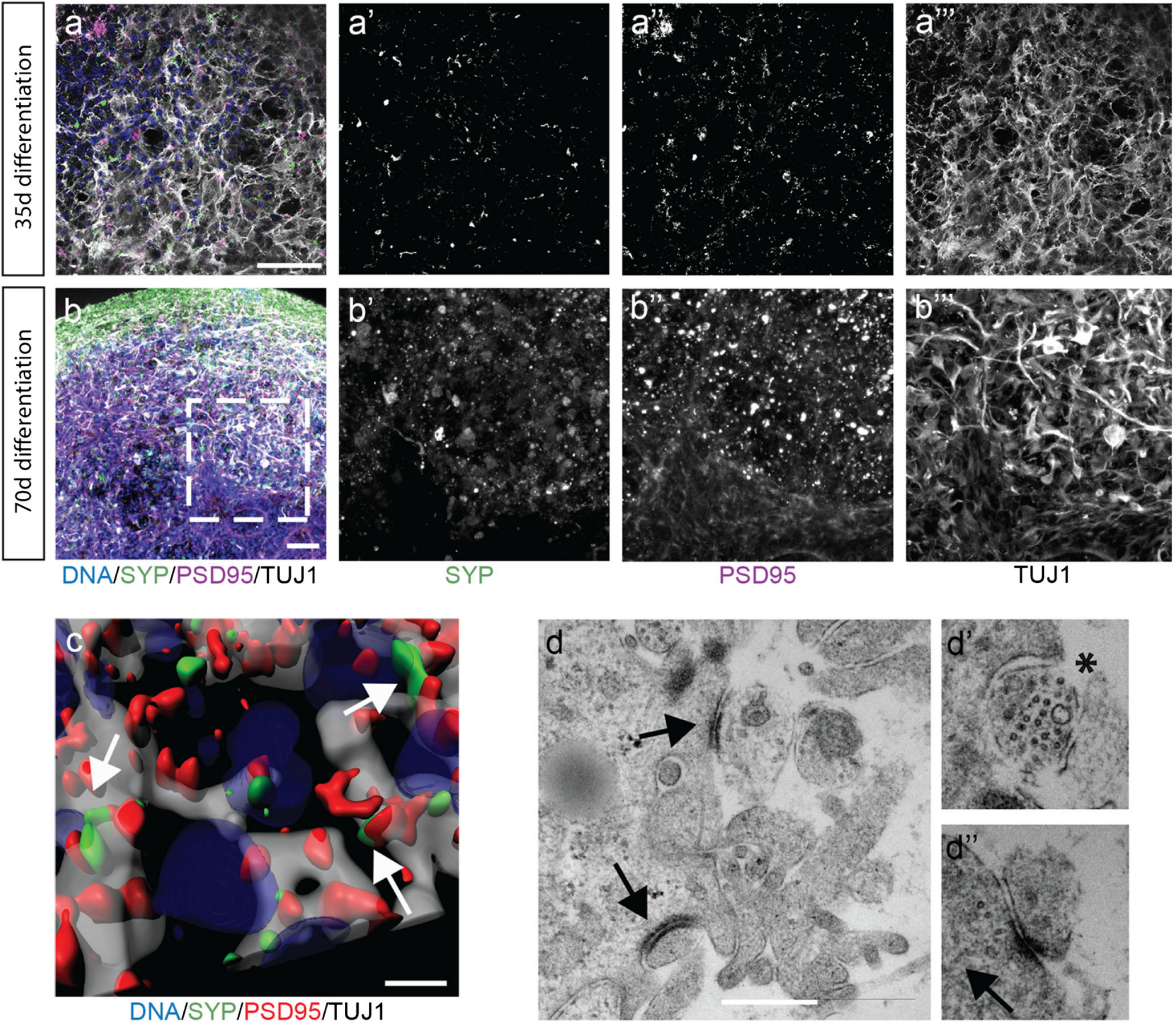
Midbrain-specific organoids express synaptic proteins. (**a**–**b**) Immunostaining of pre- and the postsynaptic markers at day 35 (**a**) and day 70 (**b**). Dashed lines indicate the region of magnification. Scale bar is 50 μm. (**c**) 3D surface reconstructions of confocal z-stacks of an organoid at day 70 of differentiation. Scale bar is 10 μm. (**d**) Representative electron micrographs of synaptic terminals from 63-day organoids. Scale bar is 500 nm

After identifying the presence of neurons and even specific neuronal subtypes on transcriptome expression levels by means of neurotransmitter staining and scRNA-seq, we investigated the actual interaction among the neuronal cells within the hMOs. We previously showed that hMOs synthesize and release the neurotransmitter dopamine (Smits et al. 2019). This already suggests the establishment of a functional neuronal network. The basic requirement for neuronal network formation is the development of synapses. Hence, we evaluated the presence of synaptic connections using the presynaptic marker SYNAPTOPHYSIN and the postsynaptic marker PSD95 in organoid sections after 35 days and 70 days of culture (Fig. 6 a and b). Both proteins were detectable in a puncta-like organization, which is expected for synapses. With a subsequent 3D surface reconstruction, we observed that the signals for SYNAPTOPHYSIN and PSD95 were localized in close proximity, forming pre- and postsynaptic puncta (Fig. 3c). To further investigate whether actual functional synaptic connections were formed in the hMOs, we used a transmission electron microscopy (TEM) approach (Fig. 3d). EM micrographs show excitatory synapses characterized by electron dense post-synaptic density proteins (full arrow) and pre-synaptic synapse (asterisks) loaded with synaptic vesicles.

### Midbrain-specific organoids develop GABAergic, glutamatergic and dopaminergic electrophysiological activity

**Fig. 7:**
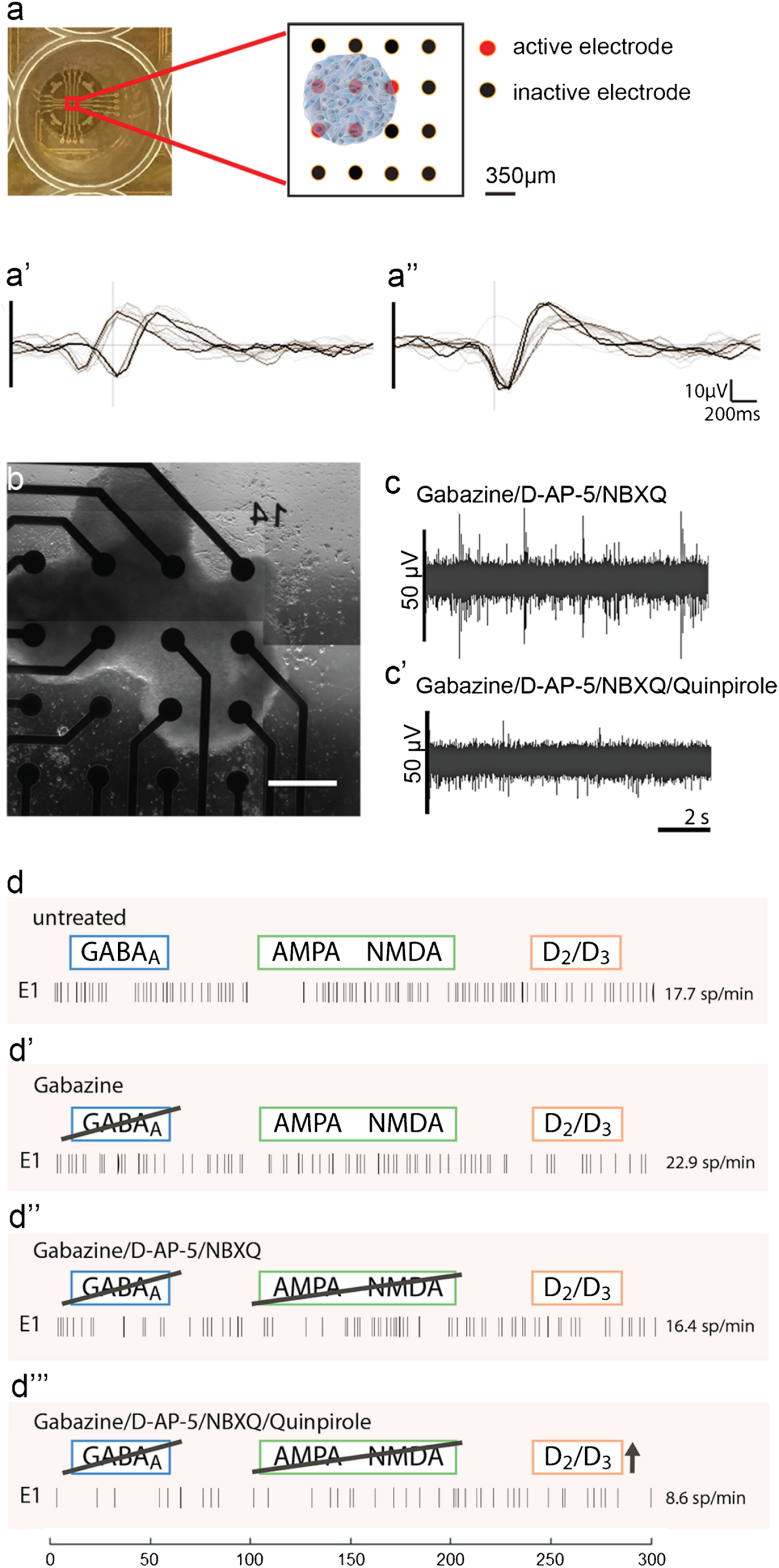
Electrophysiological activity in midbrain-specific organoids. (**a**) Representative scheme of positioned midbrain organoid on a 16-electrode array in a 48-well tissue culture plate. Examples of mono- and biphasic spikes detected by individual electrodes of a multielectrode array (MEA) system (**a’**, **a”**). (**b**) Representative image of midbrain organoid positioned on a 16-electrode array in a 48-well tissue culture plate. Scale bar is 350 μm. (**c**–**d**) Evaluation of the spontaneous activity by addressing inhibitory (blue) and excitatory (green) neurotransmitter receptors using multielectrode array (MEA) system. (**c**, **c’**) Representative raw data traces show the effect of Quinpirole in absence of inhibitory and excitatory synaptic communication. (**d**) Representative spike raster plots demonstrate effects of applied compounds

Non-invasive multielectrode array (MEA) measurements can give insights into physiological properties, like the generation of spontaneous neuronal activity of in vitro cultured, self-organized networks (Luhmann et al. 2016). As the assessment of neuronal activity is important to evaluate the functional maturation, we tested the spontaneous electrophysiological activity of hMOs by MEA measurements (Odawara et al. 2016). We measured extracellular field potentials, which are generated by action potentials. At days 50–60 of differentiation, hMOs were seeded in 48-well tissue culture MEA plates on a grid of 16 electrodes (Fig. 7 a and b). After 10–20 days of culturing, we recorded spontaneous activity, on several electrodes, over several days, in the form of mono- and biphasic spikes (Fig. 7 a’ and a”). To investigate which neuronal subtypes were functionally active in the hMOs, we applied specific drugs following a previously reported experimental design (Illes et al. 2014). We recorded spiking patterns from 24 active electrodes: in Fig. 7 c and d representative recordings of one electrode are displayed. After treating the organoids with gabazine, a GABA_A_ receptor antagonist, we detected an increase of spontaneous spiking (22.5% increase, Fig. 7d’). Following the gabazine-induced disinhibition, we applied the AMPA/Kainate-receptor antagonist NBQX and the NMDA-receptor antagonist D-AP-5. The inhibition of the excitatory neurons resulted in a 28.1% decrease of spontaneous activity (Fig. 7d”). After the inhibition of GABAergic and glutamatergic neurons in the hMOs, we added the D2/D3 receptor agonist quinpirole (Fig. 7 c’ and d”’), which resulted in a 47.8% decrease of neuronal activity. Confirming the findings displayed in Fig. 2, we conclude from these experiments that hMOs contain functional GABAergic, glutamatergic and dopaminergic neurons.

## Discussion

The in vitro human brain organoid technology has become a valuable tool allowing advances in the field of basic research as well as in translational applications (Fatehullah et al. 2016). Organoids specifically modelling the human midbrain hold great promise for studying human development and for modelling Parkinson’s disease (PD) (Jo et al. 2016; Monzel et al. 2017; Kim et al. 2019; Smits et al. 2019). In contrast to 2D monolayer cultures, hMOs can recapitulate complex interactions of midbrain dopaminergic neurons with other cell types of the central nervous system (CNS) in a 3D environment. However, human midbrain organoid research has so far focused mainly on dopaminergic neurons. In a detailed study of Borroto-Escuela et al. (2018), it has been described that released dopamine can diffuse into synaptic regions of glutamate and GABA synapses and directly affect other striatal cell types possessing dopamine receptors. Furthermore, *substantia nigra* dopaminergic neurons are directly controlled by GABAergic input (Tepper and Lee 2007). Evidences from these studies suggest that the presence of other neuronal subtypes is important to model multifactorial disease like PD. In our study, we have demonstrated that the derivation of hMOs leads to functional neuronal networks, containing different neuronal subtypes of the human midbrain. Single-cell transcriptomic data from hMOs demonstrated that there is an increased expression of neuronal-specific genes in 35 days compared with 70 days old hMOs. On the other hand, the gene-gene correlations between only neuron-specific genes increased considerably at day 70, suggesting an increased commitment of cells towards the neuronal cell fate during the course of the organoid development. This further supports the finding of a progressive maturation of post-mitotic neurons (Fig. 1c). The identification of these neuron-specific genes revealed that the genes upregulated at the earlier time point are relevant in the process of neurogenesis and neuronal migration and differentiation (EBF3 (Garcia-Dominguez et al. 2003), L1CAM (Patzke et al. 2016)). Whereas the upregulated genes at the later time point have been for instance implicated in subpopulations like GABAergic neurons (DLX1, CALB2 (Al-Jaberi et al. 2015)). This indicates a higher commitment of the cells towards their intended fate and a progressive maturation of the post-mitotic neurons within the hMOs. Furthermore, single-cell analysis of the hMOs also proved the presence of specific neuronal subtypes, like dopaminergic, glutamatergic, GABAergic and serotonergic neurons. Supporting the findings of currently published midbrain-specific organoid models (Jo et al. 2016; Qian et al. 2016; Monzel et al. 2017; Smits et al. 2019), we detected a significant upregulation of tyrosine hydroxylase (TH) within the cell population of 70 days old hMOs compared with 35 days old hMOs.

In the here presented data, we see a strong underrepresentation of neurons among recovered cells in the scRNA-seq data (see, e.g. data for 70 days of differentiation in Fig. 2a). However, this has been seen by others before and is explained by difficulties in the mechanic dissociated of complex 3D neuronal tissues into single cells. Particularly, neurons with their long and branched processes have the tendency to be lost in this process. Hence, while scRNA-seq is an excellent tool for the qualitative analysis of cell types, particularly for neural cultures, it might not be the ideal method for cell type quantification. In this context, the identification of mesenchymal cells was surprising. However, a previous study identified similar cells in human dopaminergic neuron grafts in the rodent brain (Tiklova et al. 2019). Therefore, these cells have been described as vascular leptomeningeal cells, a cell type that includes barrier forming fibroblasts. These data are consistent with our findings.

The activity of neurons and their different receptors can be analysed by the specific response to chemical compounds. It has been shown that quinpirole, a specific D2/D3 receptor agonist, suppresses the firing in hMOs (Jo et al. 2016; Monzel et al. 2017). In addition to the previously reported analyses in hMOs, we blocked inhibitory and excitatory communication, to further isolate and attribute the recorded signals to neuronal subtypes. Gabazine induces a disinhibition of GABAergic neurons, whereas NMDA-receptor and AMPA/Kainate-receptor antagonists inhibit glutamatergic excitatory communication (Illes et al. 2014). Together with the characteristic hallmarks of synapse formation (Fig. 6a–d) and the previous findings of dopamine release (Smits et al. 2019), these data confirm the presence of functional dopamine receptors in dopaminergic neurons as well as functional GABAergic and glutamatergic neurons within hMOs. As neurons do not exist in isolation in the CNS but form functional networks with other neurons and non-neuronal cells, it is important to expand our research of neurodegenerative diseases using 3D models that are able to recapitulate cell autonomous as well as non-cell autonomous aspects. Utilizing 3D cell culture models that comprise a variety of neuronal subtypes could lead to new insights into the selective vulnerabilities, which are observed in neurodegeneration. Indeed, evidence suggests that specific regulation of the excitability of dopaminergic neurons by other neuronal subtypes in the midbrain might explain their selective vulnerability in PD (Korotkova et al. 2004). This underlines the importance and the enormous potential for future disease modelling of the here described hMO model, as it contains functionally connected heterogeneous neuronal cell populations.

## Electronic supplementary material

ESM 1 (DOCX 25 kb)

ESM 2 (PDF 910 kb)

ESM 3 (PDF 472 kb)

ESM 4 (PDF 981 kb)
