# Supplementary material for: Single-cell transcriptomics reveals multiple neuronal cell types in human midbrain-specific organoids
Source: Cell Tissue Res. 2020 Jul 31;382(3):463–76. doi: 10.1007/s00441-020-03249-y (PMC7683480; doi:10.1007/s00441-020-03249-y)
Supplement: Supplementary file 1 — (DOCX 25 kb) [file 441_2020_3249_MOESM1_ESM.docx]

**Single-cell transcriptomics reveals multiple neuronal cell types in human midbrain-specific organoids**

**- Supplementary Information -**

Lisa M. Smits^1^, Stefano Magni^1#^, Kaoru Kinugawa^2#^, Kamil Grzyb^1#^, Joachim Luginbühl^3^, Sonia Sabate-Soler^1^, Silvia Bolognin^1^, Jay W. Shin^3^, Eiichiro Mori^2^, Alexander Skupin^1,4^, Jens C. Schwamborn^1^

^1^Luxembourg Centre for Systems Biomedicine (LCSB), Developmental and Cellular Biology, University of Luxembourg, Belvaux Luxembourg

^2^ Department of Future Basic Medicine, Nara Medical University, Kashihara, Nara, Japan

^3^ Division of Genomic Technologies, RIKEN Center for Life Science Technologies, Yokohama, Kanagawa, Japan

^4^University California San Diego, La Jolla, CA, USA

# Equal contribution

**Supplementary Tables**

**Supplementary Table 1**

| cell lines | Derivation conditions | Gender | Age at sampling | Genotype | Source | hiPSC ID | Figure |
| --- | --- | --- | --- | --- | --- | --- | --- |
| H1 | 3D | Female | 81 | WT | Reinhardt *et al.,* 2013 | 2.0.0.10.1.0 | 1A, B C, 2A, B, C |
| H2 | 3D | Male | n.a | WT | Alstem (iPS15) | 2.0.0.33.0.0 | 1A, B C, 2A, B, C |
| H3 | 3D | Female | n.a. | WT | Wellcome Trust Sanger Institute (Bill Skarnes) | 2.0.0.19.0.0 | 1A, B C, 2A, B, C, 3A |
| H4 | 3D | Female | cord blood | WT | Gibco (A13777) | 2.0.0.15.0.0 | 3A, B, C, D |

**Table 1:** **Cell lines used in this study to generate mfNPCs and midbrain-specific organoids.** Human mfNPCs were derived under 2D conditions from human iPSCs of different origin. hMOs were generated as described in the experimental procedures section.

**Supplementary Table 2**

| Antibody | Species | Source | Ref.-No. | Dilution |
| --- | --- | --- | --- | --- |
| Dopamine | rabbit | ImmuSmol | IS1005 | 1:500 |
| GABA | chicken | ImmuSmol | IS1036 | 1:500 |
| L-Glutamate | rabbit | ImmuSmol | IS018 | 1:500 |
| Serotonin | rabbit | ImmuSmol | IS1007 | 1:500 |
| MAP2 | mouse | Millipore | MAB3418 | 1:1000 |
| PSD-95 | rabbit | Invitrogen | 51-6900 | 1:300 |
| SYP | mouse | Abcam | ab8049 | 1:50 |
| TUJ1 | mouse | BioLegend | 801201 | 1:600 |
| TUJ1 | rabbit | Covance | PRB-435P-0100 | 1:600 |
| TUJ1 | chicken | Millipore | AB9354 | 1:600 |
| COL1A1 | rabbit | Abcam | ab34710 | 1:500 |

**Table 2: Antibodies used in this study.**

**Supplementary Table 3**

| **Stemness** | **Neuronal** | **Dopaminergic** | **Glutamatergic** | **GABAergic** | **Serotonergic** |
| --- | --- | --- | --- | --- | --- |
| SOX2 | BCL11A | NR4A2 | SLC1A1 | GAD1 | SLC6A4 |
| PAX6 | CACNA2D2 | PBX1 | SLC1A2 | GAD2 | SLC18A2 |
| HES5 | CALB2 | GRIA3 | SLC1A3 | GABARAP | *TPH1* |
| ASCL1 | *CD274* | TH | SLC17A6 | GABARAPL1 | TPH2 |
| SOX1 | CELF4 | EN1 | SLC17A7 | GABARAPL2 | FEV |
| *PAX3* | CLSTN2 | TMCC3 | GLS | *GABARAPL3* | HTR1D |
| DACH1 | DLX1 | NTM | GLS2 | ABAT | HTR1DP1 |
| LMO3 | DPYSL5 | DDC | GRIN1 |  | HTR1E |
| NR2F1 | DYNC1I1 | CAMK2N1 | GRIN2A |  | HTR1F |
| PLAGL1 | EBF3 | *ALDH1A1* | GRIN2B |  | HTR2A |
| LIX1 | FOSL2 | APP | GRIN2C |  | HTR2A-AS1 |
| HOXA2 | ISLR2 | PDZRN4 | GRIN2D |  | HTR2B |
| FOXA2 | L1CAM | PCDH10 | *GRIN3A* |  | HTR2C |
| IRX3 | *MEG3* | *MEG3* | GRIN3B |  | HTR3A |
|  | NHLH2 | ERBB4 | GRINA |  | HTR3B |
|  | NPAS4 | SLC10A4 | GRIA1 |  | HTR3D |
|  | *NPY* | BEX5 | GRIA2 |  | HTR4 |
|  | NXPH4 | FOXA2 | GRIA3 |  | HTR5A |
|  | RELN | *NPY1R* | GRIA4 |  | HTR5A-AS1 |
|  | RGMB | GPC2 |  |  | *HTR5BP* |
|  | SLC17A6 | KCNJ6 |  |  | HTR7P1 |
|  | SLC32A1 | LMX1B |  |  | HTRA1 |
|  | SST |  |  |  | HTRA2 |
|  | STMN2 |  |  |  | HTRA3 |
|  | SYNGR3 |  |  |  | HTRA4 |
|  | SYT4 |  |  |  |  |
|  | TMEM130 |  |  |  |  |
|  | VGF |  |  |  |  |
|  | VSTM2L |  |  |  |  |

**Table 3: Gene lists used in this study.** Genes that were not detected in the transcriptome are emphasised in italics.

**Supplementary Figure Legends**

**Supplementary Figure 1**

(S1a-b) Violin plots show the expression of unique feature counts (nFeature_RNA), unique molecular identifier (UMI) counts (nCount_RNA), mitochondrial transcripts proportion (percent.mt). Scatter plots shows Pearson correlation between nFeature_RNA and percent.mt. We collected 505 cells at day 35 (S1a) and 790 cells at day 70 (S1b).

**Supplementary Figure 2**

(S2a-b) Non-neuronal clusters were divided into radial glia cells (RG), mesenchymal cells (MC) and cycling progenitors (CP) subtypes. Violin plots show the distribution of expression of each marker gene at day 35 and at day 70.

**Supplementary Figure 3**

(S3a-d) UMAP plots show the gene expressions at day 35 at day 70 for markers of dopaminergic (a), GABAergic (b), glutamatergic (c), serotonergic (d) neurons. Each dot is coloured according to the expression level.
