# Supplementary figures and images for: Single-cell transcriptomics reveals multiple neuronal cell types in human midbrain-specific organoids
Source: Cell Tissue Res. 2020 Jul 31;382(3):463–76. doi: 10.1007/s00441-020-03249-y (PMC7683480; doi:10.1007/s00441-020-03249-y)

# Supplementary Figure 1

a 35d DA dif

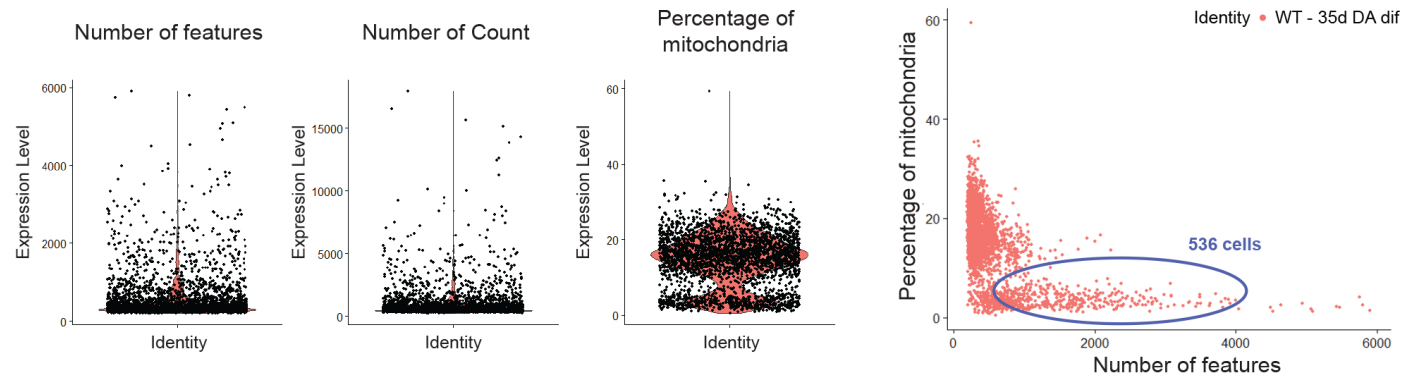

b 70d DA dif

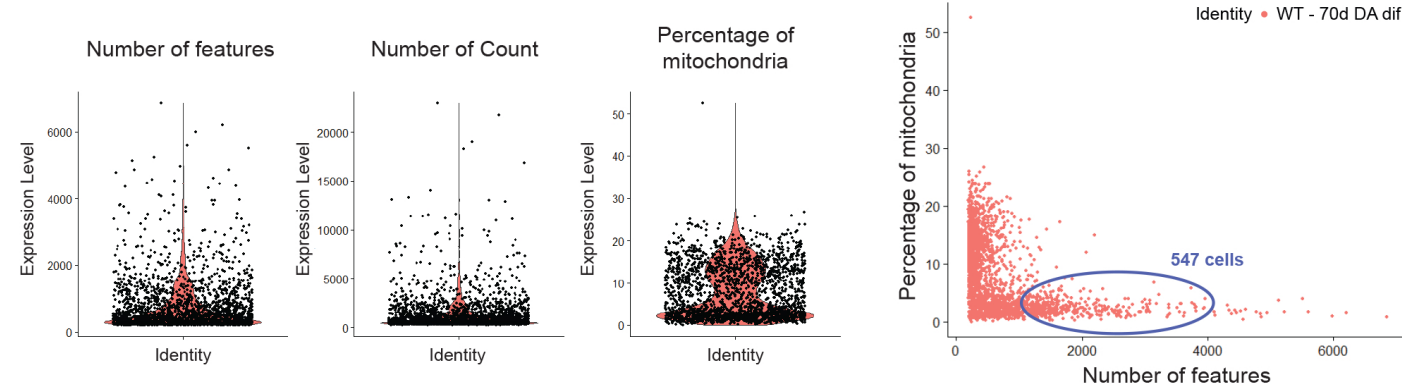

Supplement: Supplementary file 2 — (PDF 910 kb) [file 441_2020_3249_MOESM2_ESM.pdf]

Supplementary Figure 2

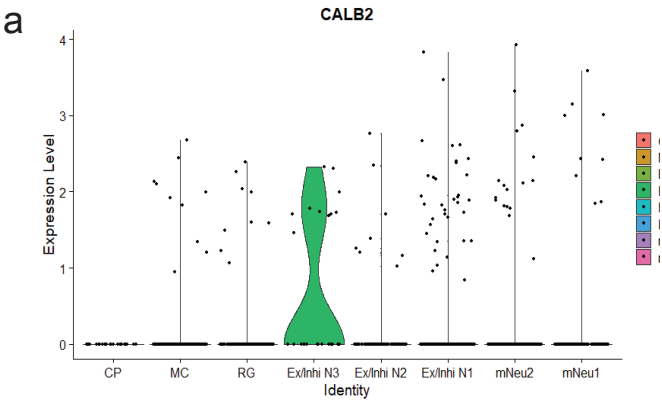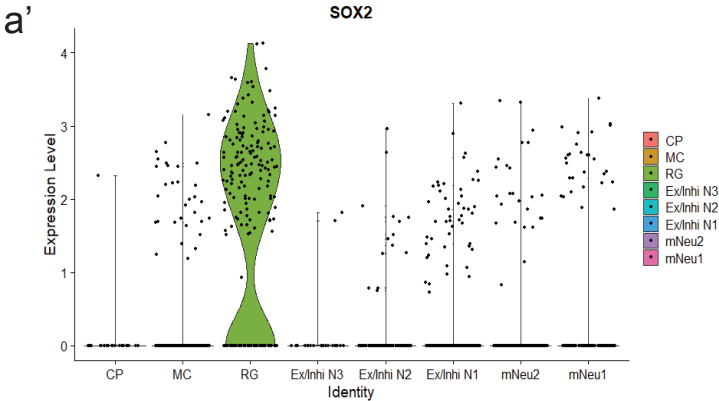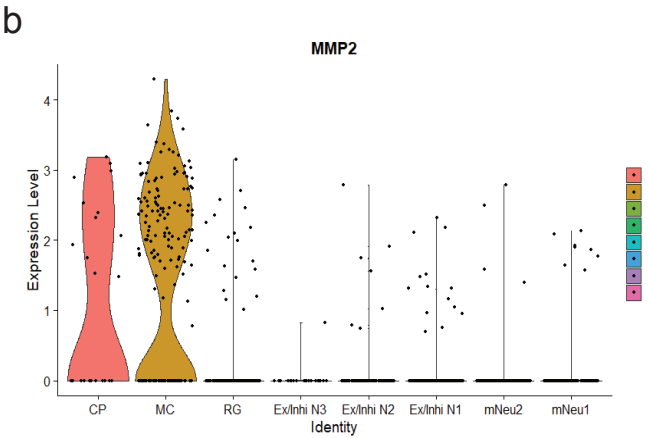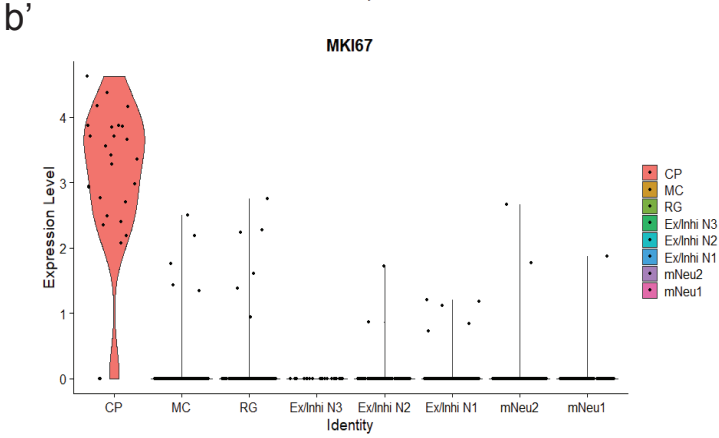

Supplement: Supplementary file 3 — (PDF 472 kb) [file 441_2020_3249_MOESM3_ESM.pdf]

Supplementary Figure 3

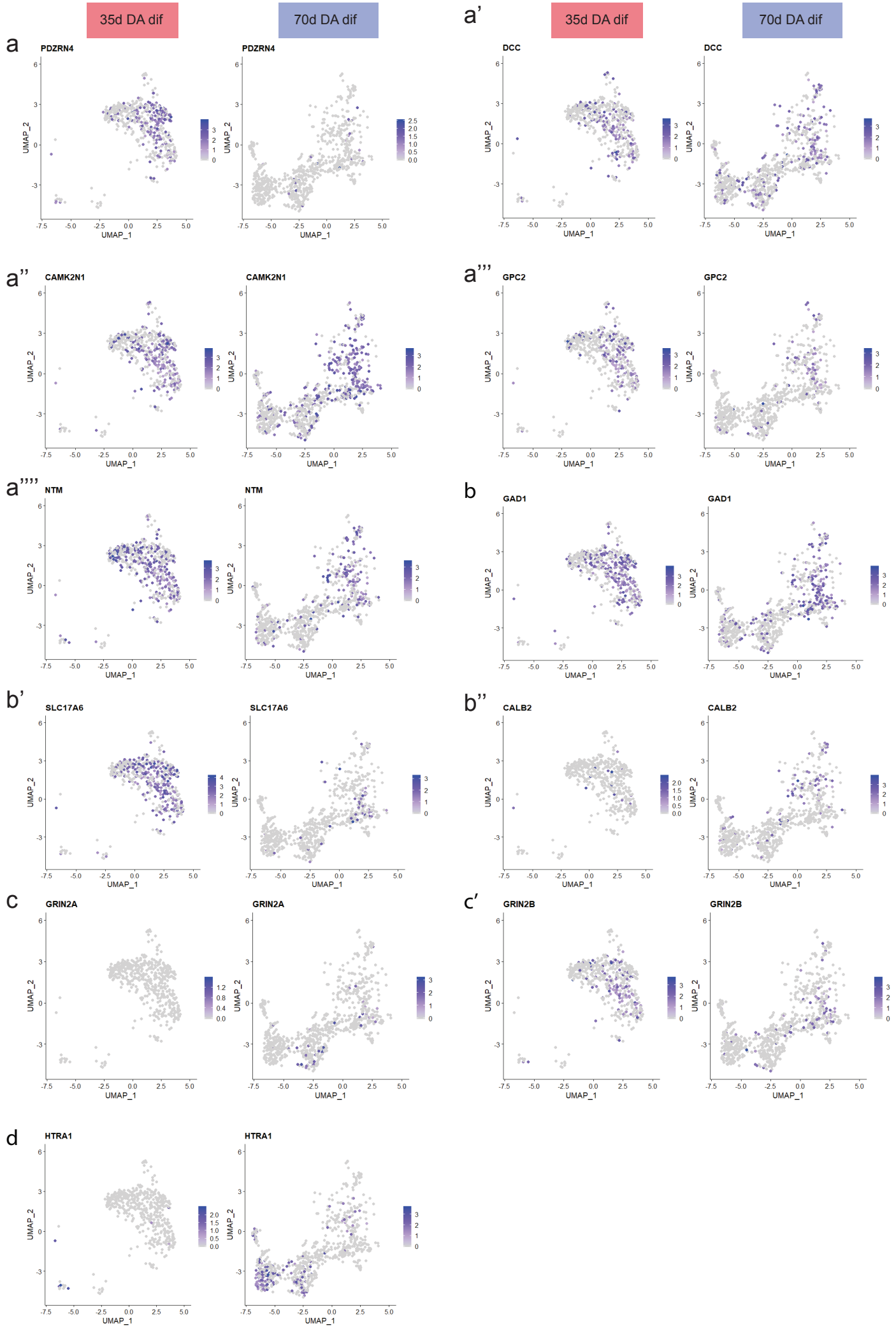

Supplement: Supplementary file 4 — (PDF 981 kb) [file 441_2020_3249_MOESM4_ESM.pdf]
